# Regulation of Chloroplast ATP Synthase Modulates Photoprotection in the CAM Plant *Vanilla planifolia*

**DOI:** 10.3390/cells11101647

**Published:** 2022-05-15

**Authors:** Hui Wang, Xiao-Qian Wang, Yi-Zhang Xing, Qing-Yun Zhao, Hui-Fa Zhuang, Wei Huang

**Affiliations:** 1 Spice and Beverage Research Institute, Chinese Academy of Tropical Agricultural Sciences, Wanning 571533, China; wanghui@catas.cn (H.W.); xyz615@catas.cn (Y.-Z.X.); qingyun_22@catas.cn (Q.-Y.Z.); 2 Kunming Institute of Botany, Chinese Academy of Sciences, Kunming 650201, China; wangxiaoqian@mail.kib.ac.cn; 3 Key Laboratory of Genetic Improvement and Quality Regulation for Tropical Spice and Beverage Crops of Hainan Province, Wanning 571533, China

**Keywords:** photosynthesis, CAM plants, photoprotection, proton gradient, cyclic electron flow

## Abstract

Generally, regulation of cyclic electron flow (CEF) and chloroplast ATP synthase play key roles in photoprotection for photosystems I and II (PSI and PSII) in C3 and C4 plants, especially when CO_2_ assimilation is restricted. However, how CAM plants protect PSI and PSII when CO_2_ assimilation is restricted is largely known. In the present study, we measured PSI, PSII, and electrochromic shift signals in the CAM plant *Vanilla planifolia*. The quantum yields of PSI and PSII photochemistry largely decreased in the afternoon compared to in the morning, indicating that CO_2_ assimilation was strongly restricted in the afternoon. Meanwhile, non-photochemical quenching (NPQ) in PSII and the donor side limitation of PSI (Y(ND)) significantly increased to protect PSI and PSII. Under such conditions, proton gradient (∆pH) across the thylakoid membranes largely increased and CEF was slightly stimulated, indicating that the increased ∆pH was not caused by the regulation of CEF. In contrast, the activity of chloroplast ATP synthase (*g*_H_^+^) largely decreased in the afternoon. At a given proton flux, the decreasing *g*_H_^+^ increased ∆pH and thus contributed to the enhancement of NPQ and Y(ND). Therefore, in the CAM plant *V. planifolia*, the ∆pH-dependent photoprotective mechanism is mainly regulated by the regulation of *g*_H_^+^ rather than CEF when CO_2_ assimilation is restricted.

## 1. Introduction

In plants, light energy absorbed by leaves drives photosynthetic electron transport in the thylakoid membranes [1,2]. During linear electron flow (LEF), electrons split from photosystem II (PSII) are transported to NADP^+^, generating the reducing power NADPH. Meanwhile, water splitting and the translocation of protons from stroma to thylakoid lumen generate proton motive force (*pmf*) across the thylakoid membranes, producing ATP through chloroplast ATP synthase. Theoretically, LEF produces a ATP/NADPH ratio of 1.29 [3,4,5], while the ATP/NADPH ratio required by primary metabolism is approximately 1.6 [4,6]. Therefore, LEF alone cannot satisfy the energy budget and plants need alternative electron flow to increase the ATP/NADPH production ratio, which is mainly accomplished by cyclic electron flow (CEF) around PSI [4,7,8,9]. During CEF, electrons are cycled back from PSI to the plastoquinone pool, which generates *pmf* without reducing NADP^+^ [10,11]. In C3 and C4 plants, the dynamic regulation of CEF adjusts the regulation of *pmf*, which is essential for photosynthesis and photoprotection under changing environmental conditions [8,12,13,14,15,16,17,18]. However, the physiological functions of CEF in CAM plants are little known.

The *pmf* across the thylakoid membranes comprise a proton gradient (ΔpH) and a membrane potential (ΔΨ), both of them can drive ATP synthesis via the chloroplast ATP synthase [19,20,21]. In addition, ∆pH is a key signal for photosynthetic regulation [15,22,23]. Particularly, ∆pH triggers the induction of non-photochemical quenching (NPQ) in PSII, which is critical to dissipation of excess light energy and photoprotection of PSII [12,24,25,26]. Furthermore, ∆pH adjusts the redox state of PSI and prevents PSI photoinhibition [12,14,27,28,29,30]. Once the buildup of ∆pH was suppressed under high light, the induction of NPQ would be suppressed and PSI would be over-reduced, leading to detrimental photodamage to PSII and PSI [22,25]. If the formation of ∆pH was too high, the plastoquinone oxidation at the cytochrome (Cyt) *b*_6_/*f* complex was down-regulated, restricting the operation of LEF and CO_2_ assimilation [31,32]. Therefore, ∆pH should be regulated finely to optimize photosynthetic CO_2_ assimilation and photoprotection.

Generally, the buildup of ∆pH is controlled by photosynthetic electron flows and chloroplast ATP synthase [11,15]. The impairment of CEF in proton gradient regulator5 (*pgr5*) mutant of *Arabidopsis thaliana* reduces ∆pH formation under high light, resulting in severe photoinhibition of PSI and PSII [12,14,25]. In *hope2* and *cfq* mutants of *A. thaliana*, the activity of the chloroplast ATP synthase (*g*_H_^+^) largely increased, resulting in decreased ∆pH formation and thus inducing severe loss of PSI activity under high light or fluctuating light [28,29]. In C3 plants, stimulation of CEF and down-regulation of *g*_H_^+^ simultaneously contribute to the increase in ∆pH when CO_2_ assimilation was restricted by environmental stresses, such as low CO_2_ concentration [33,34], drought [35,36,37], low temperature [38,39], and fluctuating light [27,40,41,42,43]. By comparison, when CO_2_ assimilation was restricted by low CO_2_ concentration in the model C4 plant maize, the increase of ∆pH was caused by the decrease of *g*_H_^+^ rather than stimulating CEF [34]. Therefore, C3 and C4 plants have different strategies for regulation of ∆pH formation when CO_2_ assimilation was restricted. Within the first seconds after transition from dark or low light to high light, the relatively low *g*_H_^+^ and CEF stimulation contributed to the rapid formation of ∆pH, favoring photoprotection for PSI and PSII in the CAM plant *Bryophyllum pinnatum* [44], which was similar to the phenomenon in C3 plants. In contrast to C3 and C4 plants, crassulacean acid metabolism (CAM) plants usually close stomata in daytime to survive drought habitat [45]. At daytime, CO_2_ released from malic acid is assimilated by the Calvin–Benson cycle. Owing to the gradual consumption of malic acid at daytime, the chloroplast CO_2_ concentration decreased in the afternoon [46,47], restricting photosynthetic light use efficiency [48]. Under such conditions, a high level of ∆pH should be formed to protect photosynthetic apparatus against photoinhibition. However, the regulatory mechanism of ∆pH formation is still poorly understood in CAM plants.

In this study, light response changes of PSI, PSII, ΔpH, and *g*_H_^+^ were measured in the morning and in the afternoon for leaves of the CAM plant *Vanilla planifolia*. The aims are to: (1) Explore the performance of CEF when CAM photosynthesis is restricted; and (2) assess how CAM plants regulate ∆pH when light use efficiency is restricted. Our results indicated that in *V. planifolia* CEF/LEF ratio slightly increased when LEF was restricted in the afternoon. Meanwhile, the ∆pH level largely increased to trigger photoprotection, which was predominantly caused by the decrease of *g*_H_^+^. Therefore, regulation of *g*_H_^+^ plays a more important role in ∆pH formation than CEF in the CAM plant *V. planifoli* upon restriction of photosynthetic CO_2_ assimilation. Regulation of *g*_H_^+^ is likely a common strategy used by C3, C4, and CAM plants to modulate photoprotection responding to environmental conditions.

## 2. Materials and Methods

### 2.1. Plant Materials and Growth Conditions

*Vanilla planifolia* Andrews is a famous spice plant with high commercial value. As a CAM plant native to tropical forests in Mexico and Central America, *V. planifolia* is adaptive to hot, humid, and shade environment. The studied plants of *V. planifolia* were cultivated in Spice and Beverage Research Institute, Chinese Academy of Tropical Agricultural Sciences (18°70′ E, 110°18′ N, altitude 10 m). The shade condition was controlled using two-layer polyester shade net, and the maximum light intensity exposed to leaves was approximately 200 μmol photons m^−2^ s^−1^. We conducted photosynthetic measurements on youngest fully expanded but not-senescent leaves in November in 2021. During this period, the day/night air temperatures were approximately 33/25 °C and the relative air humidity approximately 80%.

### 2.2. PSI and PSII Measurements

Our previous study indicated that for leaves of *V. planifolia*, the light use efficiency was maximized in the morning (a.m. 10:00–12:00) but was restricted in the afternoon (p.m. 13:00–17:00) [48]. Therefore, at these two time slots, a Dual-PAM 100 measuring system (Heinz Walz, Effeltrich, Germany) was used to record PSI and PSII parameters at approximately 30 °C. After dark-adaptation for 30 min, leaves were illuminated at 923 μmol photons m^−2^ s^−1^ for 15 min to reach steady state photosynthesis. Afterward, light response curves were measured. PSI and PSII parameter were calculated as follows [49,50]: The quantum yield of PSI photochemistry, Y(I) = (*P*_m_*’* − *P*)/*P*_m_; the quantum yield of PSI non-photochemical energy dissipation due to donor side limitation, Y(ND) = *P*/*P*_m_; the quantum yield of PSI non-photochemical energy dissipation due to acceptor side limitation, Y(NA) = (*P*_m_ − *P*_m_*’*)/*P*_m_; the quantum yield of PSII photochemistry, Y(II) = (*F_m_’* − *F_s_*)/*F_m_’*; non-photochemical quenching, NPQ = (*F_m_* − *F_m_’*)/*F_m_’*; the quantum yield of non-regulatory energy dissipation in PSII, Y(NO) = *F_s_*/*F_m_*; the maximum efficiency of the open PSII centers in the light, *F*_v_′/*F*_m_′ = (*F_m_’* − *F_o_’*)/*F_m_’*; the coefficient of photochemical quenching based on the “puddle model” of PSII, qP = (*F_m_’* − *F_s_*)/(*F_m_’* − *F_o_’*); the coefficient of photochemical quenching based on the “lake model” of PSII, qL = qP × *F_o_’*/*F_s_*. *F_o_’* is estimated using the following equation, *F_o_’* = *F_o_*/(*F*_v_/*F*_m_ + *F_o_*/*F_m_’*) [51]. *P*_m_, *F*_m_, and *F*_o_ were measured after dark adaptation for 30 min. *P*_m_*’*, *P*, *F*_m_*’*, and *F*_s_ were measured after exposure to each light intensity for 3 min.

The photosynthetic electron transport rates through PSI and PSII were calculated as follows: ETRI = PAR × Y(I) × 0.84 × dI; ETRII = PPFD × Y(II) × 0.84 × dII. PPFD is the photosynthetically active radiation, and 0.84 is the assumed light absorption of incident irradiance. Based on the assumption that the role of CEF is negligible under very low light intensity [52], the values of dI and dII were calculated using the values of Y(I) and Y(II) under a low light of 59 μmol photons m^−2^ s^−1^ in the morning according to the method of [37]. dI and dII were 0.487 and 0.513, respectively. Furthermore, these estimated values of dI and dII were also used for the calculation of ETR in the afternoon. The apparent rate of CEF was estimated by subtracting ETRII from ETRI [30,37].

### 2.3. Electrochromic Shift Measurement

A Dual-PAM 100 measuring system equipping a P515/535 emitter-detector module was used to measure the electrochromic shift signals (ECS) [53,54]. After photosynthetic induction at 923 μmol photons m^−2^ s^−1^ for 15 min, the ECS were recorded after illumination at each light intensity for 3 min. ECS dark interval relaxation kinetics were recorded to calculate the ΔpH and *g*_H_^+^ [35,55,56].

### 2.4. Statistical Analysis

All data are displayed as mean values of five leaves from five independent plants. T-test was used to determine whether significant differences existed between different treatments (*α* = 0.05).

## 3. Results

**Figure 1:**
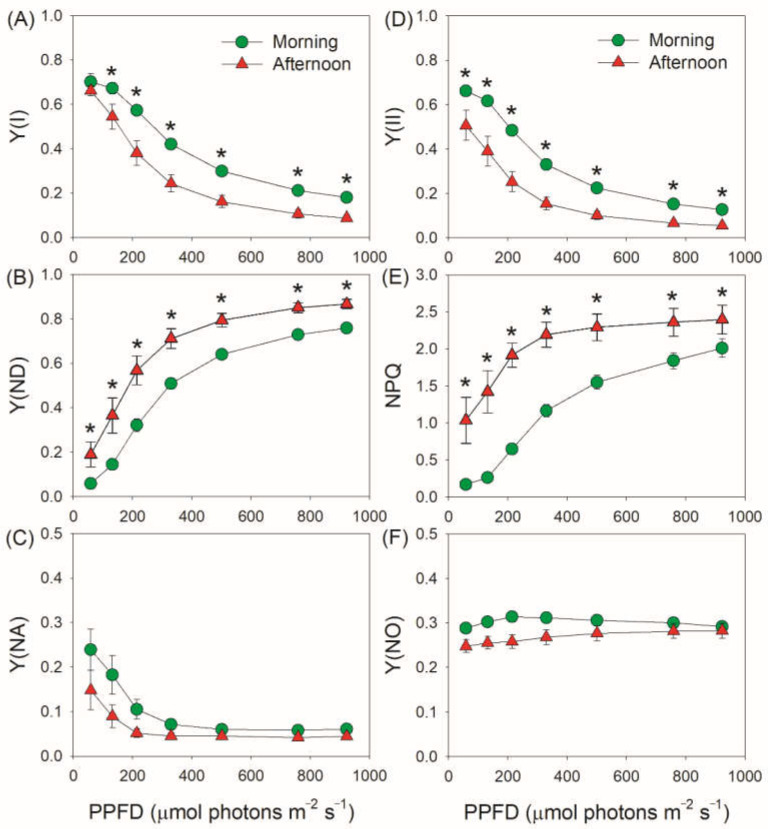
Light intensity dependence of PSI and PSII parameters for leaves of *Vanilla planifolia* measured in the morning and in the afternoon. Y(I) (**A**), the quantum yield of PSI photochemistry; Y(ND) (**B**), the quantum yield of PSI non-photochemical energy dissipation due to donor side limitation; Y(NA) (**C**), the quantum yield of PSI non-photochemical energy dissipation due to acceptor side limitation; Y(II) (**D**), the quantum yield of PSII photochemistry; NPQ (**E**), non-photochemical quenching in PSII; Y(NO) (**F**), the quantum yield of non-regulatory energy dissipation in PSII. Data are means ± SE (*n* = 5). Asterisk indicates a significant difference between morning and afternoon.

To examine the effect of restriction of CO_2_ assimilation on PSI and PSII performances, we first measured the light response changes of PSI and PSII parameters in the morning and in the afternoon for leaves of *V. planifolia* (Figure 1). The quantum yield of PSI photochemistry (Y(I)) gradually decreased with the increase in light intensity, and the values of Y(I) in the morning were higher than those in the afternoon (Figure 1A). Concomitantly, the PSI donor side limitation (Y(ND)) gradually increased in both samples with higher values in the afternoon (Figure 1B), resulting in similar PSI acceptor side limitation (Y(NA)) between morning and afternoon (Figure 1C). The effective quantum yield of PSII photochemistry (Y(II)) gradually decreased with the increasing light intensity (Figure 1D). In the afternoon, Y(II) significantly decreased at any light intensity when compared with in the morning. Concomitantly, non-photochemical quenching (NPQ) was significantly enhanced in the afternoon to dissipate the excess light energy (Figure 1E). As a result, the quantum yield of non-regulatory energy dissipation in PSII (Y(NO)) was maintained at low level (Figure 1F), diminishing the risk of PSII photoinhibition. Therefore, although Y(I) and Y(II) were largely restricted in the afternoon, PSI over-reduction was completely prevented and PSII photoprotection was well performed.

**Figure 2:**
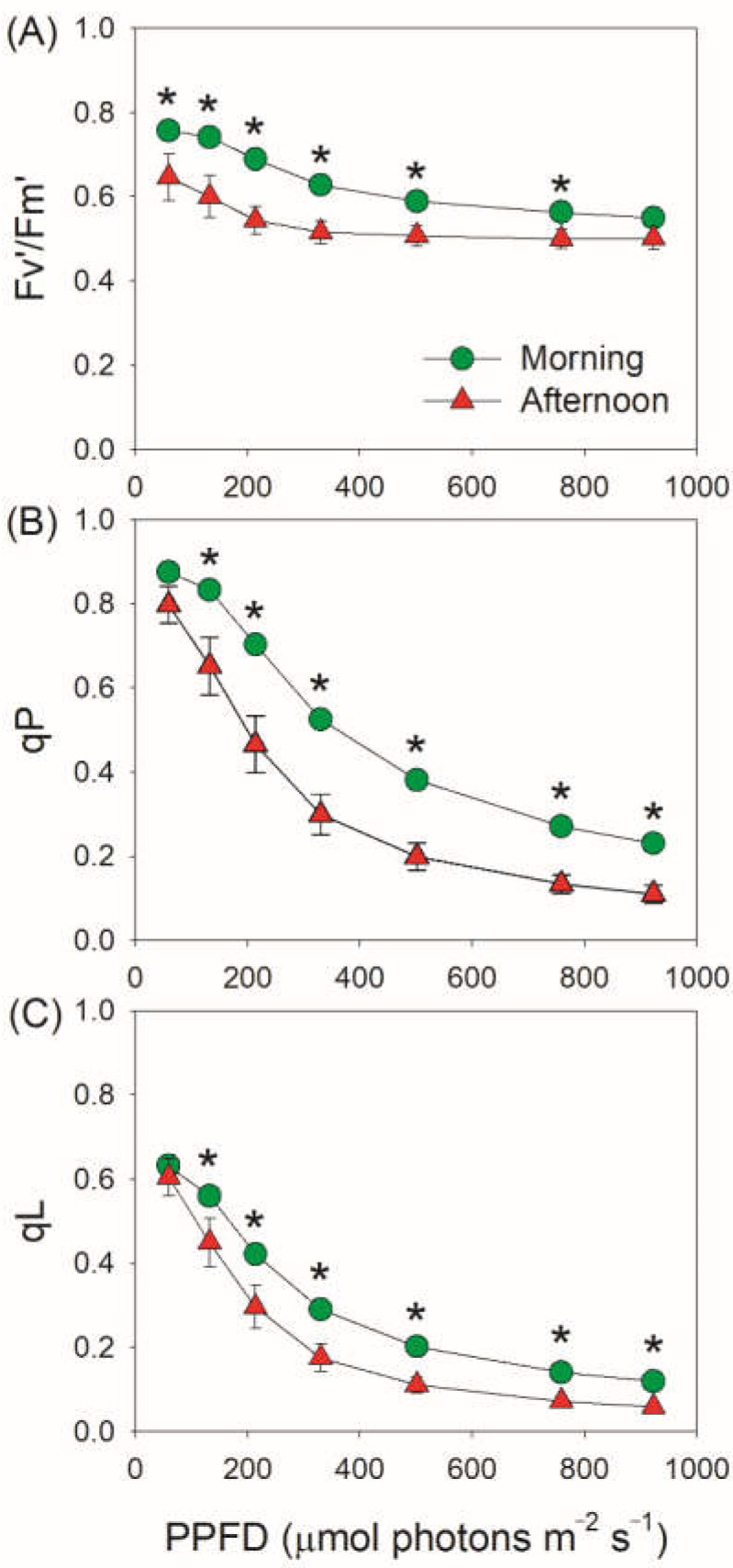
Light intensity dependence of *F*_v_′/*F*_m_′ (**A**), qP (**B**) and qL (**C**) for leaves of *Vanilla planifolia* measured in the morning and in the afternoon. *F*_v_′/*F*_m_′, the maximum efficiency of the open PSII centers in the light; qP, coefficient of photochemical quenching based on the “puddle model” of PSII; qL, coefficient of photochemical quenching based on the “lake model” of PSII. Data are means ± SE (*n* = 5). Asterisk indicates a significant difference between morning and afternoon.

**Figure 3:**
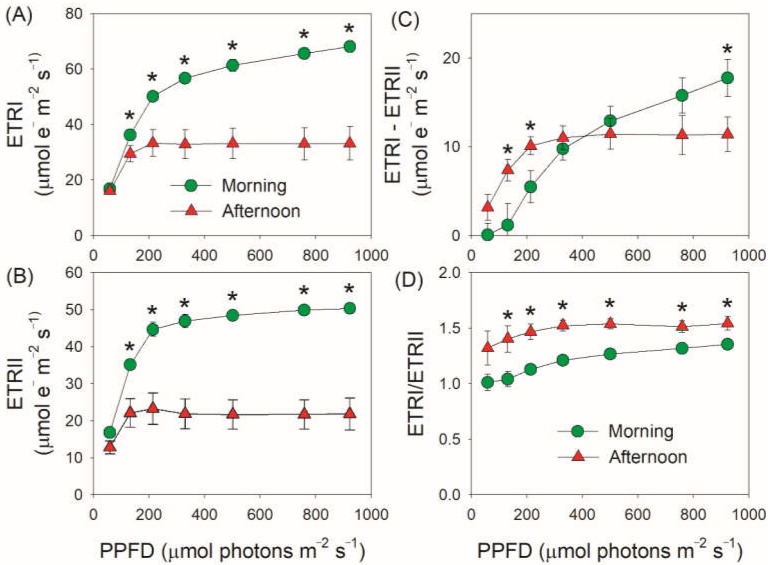
Light intensity dependence of electron transport rates for leaves of *Vanilla planifolia* measured in the morning and in the afternoon. ETRI (**A**), electron transport rate through PSI; ETRII (**B**), electron transport rate through PSII; ETRI–ETRII (**C**), estimated rate of cyclic electron flow; ETRI/ETRII (**D**), an indicator reflecting the contribution of cyclic electron flow to total photosynthetic electron flow. Data are means ± SE (*n* = 5). Asterisk indicates a significant difference between morning and afternoon.

The value of Y(II) is the product of *F*_v_′/*F*_m_′ and qP. *F*_v_′/*F*_m_′ represents the maximum efficiency of the open PSII centers in the light, and qP represents the coefficient of photochemical quenching based on the “puddle model” of PSII. We found that the decreased Y(II) in the afternoon was mainly caused by the decrease in qP rather than the change in *F*_v_′/*F*_m_′ (Figure 2A,B). Furthermore, we found that the coefficient of photochemical quenching based on the “lake model” of PSII (qL) also decreased in the afternoon (Figure 2C). These results indicated that the PSII reaction centers were closed to a higher extent in the afternoon. Based the data of Y(I) and Y(II), photosynthetic electron transport rate through PSI (ETRI) and PSII (ETRII) and the rate of CEF were calculated. Under light intensities above 200 μmol photons m^−2^ s^−1^, ETRI and ETRII largely decreased in the afternoon (Figure 3A,B). Meanwhile, the value of ETRI–ETRII was enhanced under light intensities below 330 μmol photons m^−2^ s^−1^ but stagnated above this light intensity (Figure 3C). Furthermore, the ETRI/ETRII ratio was enhanced in the afternoon (Figure 3D). The activation of CEF is dependent on dynamic thylakoid stacking and *pgr5*-pathway [30]. The light response changes of ETRI–ETRII and ETRI/ETRII strongly indicated the activation of CEF under high light [30]. These results indicated that LEF and CEF responded differently to the restriction of CO_2_ assimilation in the CAM plant *V. planifolia*. It should be noted that this method of estimating CEF has some limits. First, light intensity can affect the partitioning of light between PSI and PSII. Second, Y(I) will be increased if electron supply to PSI is throttled up to a certain extent, as indicated by the lower Y(II) and higher Y(I) under moderate light in *pgr1* mutant compared with WT plants of *Arabidopsis thaliana* [57,58].

**Figure 4:**
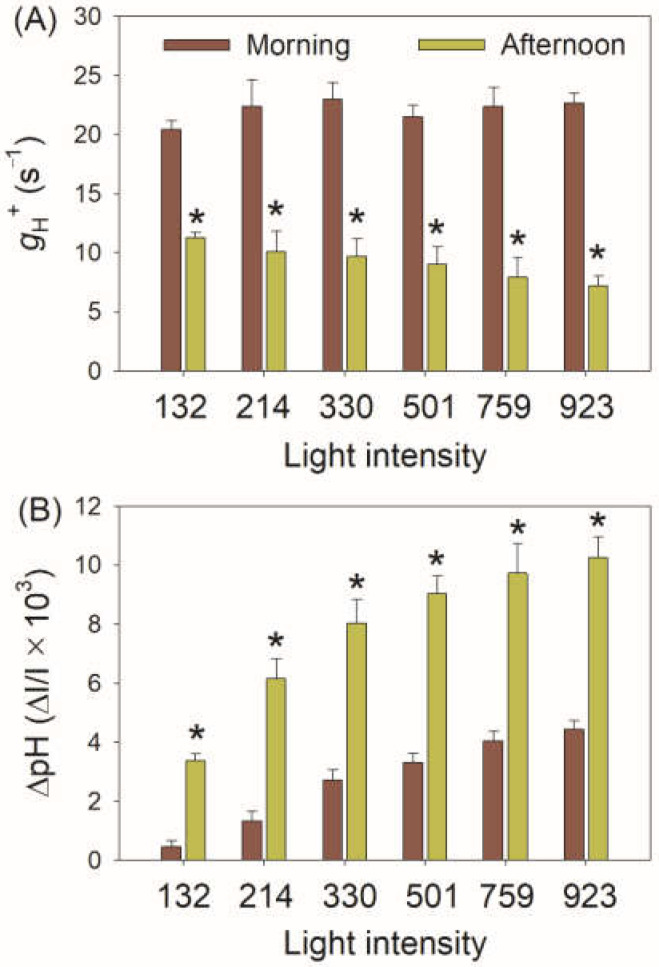
Light intensity dependence of *g*_H_^+^ (**A**) and ∆pH (**B**) for leaves of *Vanilla planifolia* measured in the morning and in the afternoon. *g*_H_^+^, the conductivity of the chloroplast ATP synthase to protons; ∆pH, proton gradient across the thylakoid membranes. Data are means ± SE (*n* = 5). Asterisk indicates a significant difference between morning and afternoon.

**Figure 5:**
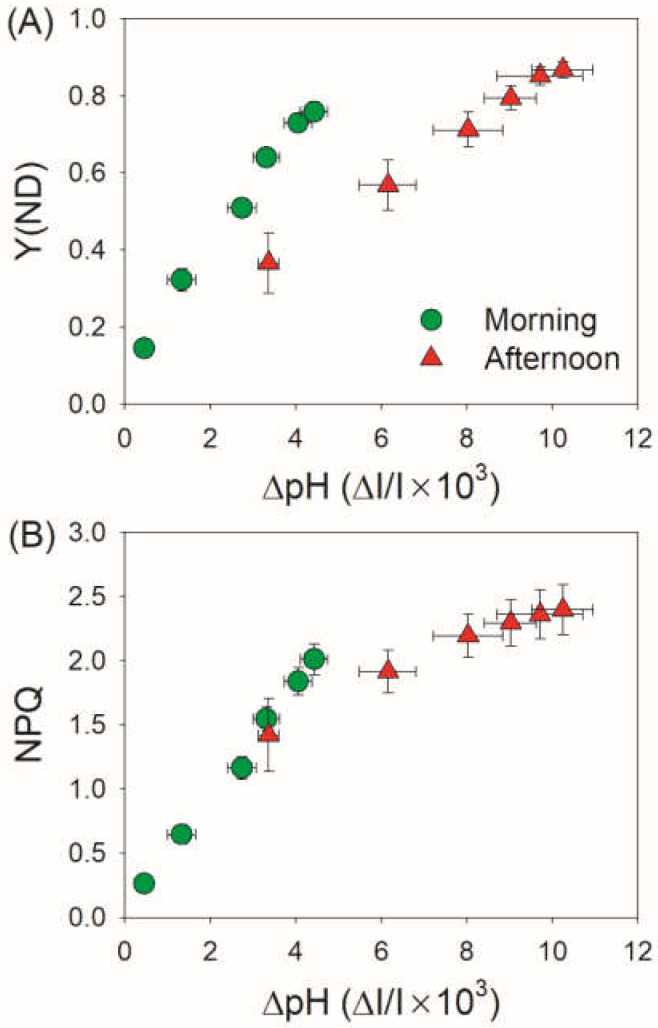
Changes in (**A**) Y(ND) and (**B**) NPQ as a function of ∆pH for leaves of *Vanilla planifolia* measured in the morning and in the afternoon. Data are means ± SE (*n* = 5).

Because the activity of chloroplast ATP synthase (*g*_H_^+^) and ∆pH are key factors for photosynthetic regulation when CO_2_ assimilation is restricted, we next measured the electrochromic shift signals for leaves of *V. planifolia*. With increasing light intensity, *g*_H_^+^ was maintained stable in the morning but gradually decreased in the afternoon with low values (Figure 4A). Furthermore, the value of *g*_H_^+^ in the morning was 2–3-fold than that in the afternoon. Meanwhile, the ∆pH formation was largely enhanced in the afternoon (Figure 4B). Therefore, when CO_2_ assimilation was restricted in the afternoon, the activity of chloroplast ATP synthase was suppressed and the acidification of thylakoid lumen was increased. The increase of ∆pH triggered the induction of NPQ and PSI donor side regulation, as indicated by positive effects of ∆pH on NPQ and Y(ND) (Figure 5). Furthermore, when CO_2_ assimilation was restricted in the afternoon, ∆pH was formed to much higher levels than that required for the NPQ induction and PSI donor side regulation.

**Figure 6:**
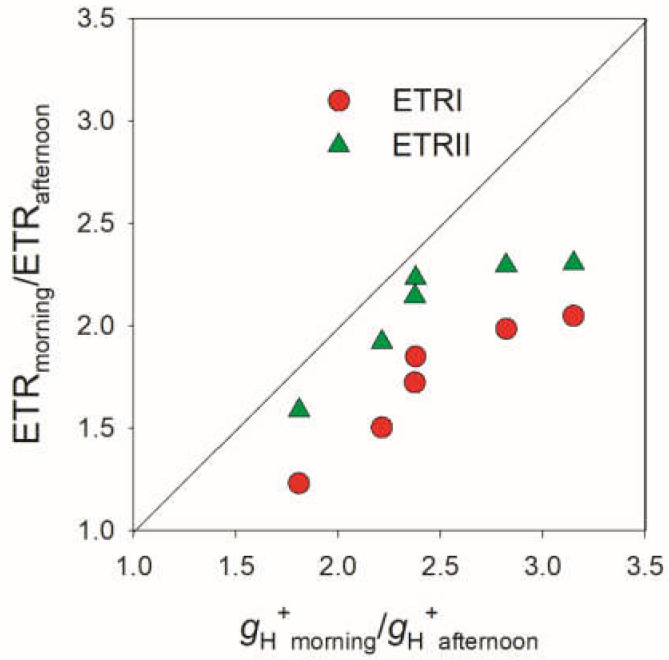
Changes in the decrease amplitudes of ETRI and ETRII in the afternoon as a function of the decrease amplitude of *g*_H_^+^ for leaves of *Vanilla planifolia*. Light response data from Figure 3 and Figure 4 were used to calculate the decrease amplitudes of ETRI, ETRII, and *g*_H_^+^ in the afternoon when compared with those in the morning.

To explore why the lower proton flux activity (ETRI and ETRII values) in the afternoon was accompanied by higher ∆pH, we compared the decreased amplitude of ETRI, ETRII, and *g*_H_^+^ in the afternoon. As shown in Figure 6, the decreased amplitude of *g*_H_^+^ in the afternoon was stronger than that of ETRI and ETRII. These results indicated that the large decrease in *g*_H_^+^ in the afternoon compensated for the decreases in LEF and CEF under high light. In the afternoon, ETRII and CEF were saturated under a moderate light of 132 μmol photons m^−2^ s^−1^ (Figure 3B,C), while ∆pH gradually increased with light intensity (Figure 4B). These results indicated that the increased ∆pH under high light was not caused by the changes in ETRII and CEF. Therefore, the lowering of *g*_H_^+^ was the primary cause of increased ∆pH when CO_2_ assimilation under high light was restricted in *V. planifolia*.

## 4. Discussion

During photosynthesis, photosynthetic electron transport in thylakoids produces ATP and NADPH for CO_2_ fixation in chloroplast stroma [20,59]. PSI and PSII are two key components of photosynthetic electron transport chains, requiring finely balanced photoprotection for them [60,61,62]. If PSI was photodamaged, both LEF and CEF were restricted, CO_2_ assimilation and photoprotection would be suppressed, impairing plant growth [14,63,64,65]. Alternatively, if PSII photoinhibition occurred, LEF would be reduced due to the decreased splitting of water, decreasing the regeneration of ATP and NADPH and thus declining CO_2_ assimilation [25,66,67]. When CO_2_ assimilation is restricted in C3 and C4 higher plants, ∆pH is the key signal for photoprotection against excess light energy [33,34,35,38]. However, the role of ∆pH formation in photoprotection for PSI and PSII in CAM plants is little known.

We found that ETRII largely decreased in the afternoon for leaves of the CAM plant *V. planifolia* (Figure 3B), indicating that CO_2_ assimilation was restricted. In the afternoon, the stomatal closure in *V. planifolia* restricted CO_2_ diffusion from air to chloroplast [48]. Therefore, the decreased ETRII in the afternoon was caused by the drying up of the malic acid pool [46,47]. At this time, Y(ND) largely increased and Y(NA) was maintained at low levels (Figure 1B,C), suggesting that PSI was highly oxidized and PSI over-reduction was prevented. The prevention of PSI over-reduction diminished the production of reactive oxygen species around PSI reaction centers and thus avoided PSI photoinhibition [14,68,69]. It has been documented that the steady-state PSI redox state under high light is largely regulated by ∆pH [11,13,28]. A high ∆pH is essential to prevent PSI over-reduction under high light at donor and acceptor sides [69]. In donor side regulation, a high ∆pH restricts Qo-site activity in the Cyt *b*_6_/*f* complex, such lumen pH-dependent “photosynthetic control” slows down the electron flow from PSII to PSI and thus increases Y(ND). In acceptor side regulation, a high ∆pH regulates the ATP/NADPH production ratio for CO_2_ assimilation and photorespiration, which facilitates the electron sink downstream of PSI and thus prevents PSI over-reduction [70]. In rice (*Oryza sativa*) mutants defective in the PGR5/PGRL1-dependent CET or both CET pathways, CO_2_ is significantly disturbed and acceptor side regulation is weakened [71,72]. As shown in Figure 5A, Y(ND) was tightly correlated to the ∆pH formation, but there were two distinct degrees of photosynthetic control in morning vs. afternoon. In the morning, a relatively low ∆pH weakened photosynthetic control at the Cyt *b*_6_/*f* complex, but a high Y(ND) was achieved due to the higher light use efficiency. When CO_2_ assimilation was restricted in the afternoon, a higher ∆pH-dependent photosynthetic control was necessary to maintain the high level of Y(ND). Therefore, ∆pH-dependent photosynthetic control was more important in photoprotection for PSI when photosynthesis of *V. planifolia* was restricted in the afternoon.

In addition to regulate PSI redox state, ∆pH also induces NPQ in light-harvesting complex II and the PSII core, which is essential for PSII photoprotection [13,25,26,73]. In the CAM plant *V. planifolia*, NPQ was up-regulated with the suppression of CO_2_ assimilation (Figure 1E). Such enhancement of NPQ decreased the production of reactive oxygen species in PSII. Because reactive oxygen species produced within PSII inhibits the repair of PSII [74,75,76,77], the ∆pH-dependent induction of NPQ in *V. planifolia* facilitated the repair of PSII. Furthermore, a high ∆pH could sequester Ca^2+^ in the lumen through a Ca^2+^/H^+^ antiport, and a high Ca^2+^ concentration in the lumen could protect the oxygen-evolving complex. Once ∆pH formation under high light was suppressed, photodamage of oxygen-evolving complex further aggravated PSII photoinhibition [25,60,78]. Interestingly, we observed that *V. planifolia* generated a much higher ∆pH than that required by the induction of the maximum NPQ when CO_2_ assimilation was restricted (Figure 5B). Therefore, the much higher ∆pH level in the afternoon might protect the oxygen-evolving complex in PSII. Taking together, a high ∆pH is essential for photoprotection of PSII when photosynthesis of *V. planifolia* was restricted in the afternoon.

Both LEF and CEF pump electrons from the chloroplast stroma into the thylakoid lumen and thus induce the ∆pH formation [11,59]. In addition, the splitting of water in LEF generates H+ in the lumen of thylakoid. Therefore, the formation of ∆pH is in general primarily dependent on LEF rather CEF because LEF is usually higher than CEF [33,34,79]. However, when CO_2_ assimilation and LEF are restricted under environmental stresses in C3 plants, CEF plays an essential role in the ∆pH formation under high light [43,80,81]. For example, the impairment of CEF in *A. thaliana pgr5* and *ndh* mutants decreased the ∆pH formation under high light [53,82]. In the C3 resurrection plant *Paraboea rufescens*, CEF was highly stimulated to a much higher level than LEF when leaves were dehydrated [37]. By comparison, promoting CEF played a minor role in the enhancement of ∆pH in the model C4 maize when CO_2_ assimilation is restricted [34], although CEF was essential for the normal photosynthesis at ambient O_2_ concentration in C4 plants [17,83,84]. This different performance of CEF between C3 and C4 plants is mainly caused by the capacity of photorespiration between them [34]. In the studied CAM plant *V. planifolia*, CEF was stimulated under low and moderate light intensities but was suppressed under high light once CO_2_ assimilation was restricted (Figure 3C). Meanwhile, the CEF/LEF ratio increased at all light intensities (Figure 3D), suggesting the contribution of CEF to ∆pH formation was enhanced. Such CEF stimulation favored ∆pH-dependent photoprotective mechanisms and increased the ATP/NADPH production ratio for photorespiration [4,11]. Therefore, CEF was required to facilitate photosynthetic regulation in the CAM plants, similar to the phenotype in C3 plants but different from that in C4 plants.

The other important factor for optimizing the formation of ∆pH is the proton conductivity of chloroplast ATP synthase, as indicated by *g*_H_^+^ measured by electrochromic shift signals in this study [28,29,31,33]. Once ATP synthase accumulation was largely reduced in tobacco, the lowering of *g*_H_^+^ generated a much higher ∆pH and impaired plant growth [31]. On the other hand, the increasing of *g*_H_^+^ suppressed the formation of ∆pH in *A. thaliana hope2* and *cfq* mutants, resulting in severe PSI photoinhibition under high light or fluctuating light [28,29]. In C3 plants, *g*_H_^+^ decreased under drought [35,85], low CO_2_ concentration [33,34], and chilling temperature [38], which is critical for the increasing of ∆pH and photoprotection. When CO_2_ assimilation was suppressed, the decrease of *g*_H_^+^ was stronger than that of LEF in C3 plant *Brassica rapa* but was equal to that of LEF in maize [34]. In this study, we here documented that *g*_H_^+^ decreased greater than ETRI and ETRII in the CAM plant *V. planifolia* when CO_2_ assimilation was suppressed in the afternoon (Figure 6). When illuminated at a saturating light of 923 in the afternoon, both LEF and CEF were suppressed (Figure 3B,C) but ∆pH was highly increased (Figure 4B). Therefore, the regulation of *g*_H_^+^ plays a more important role than CEF in the regulation of ∆pH formation when CO_2_ assimilation is restricted in the CAM plant *V. planifolia*.

## 5. Conclusions

In this study, we studied how the CAM plant *V. planifolia* regulates the formation of ∆pH according to the change in light use efficiency. We found that when light use efficiency was restricted in the afternoon, *g*_H_^+^ largely decreased and CEF activity slightly increased, both of which contributed to the enhancement of ∆pH formation. Subsequently, ∆pH-dependent photoprotective mechanisms were highly activated to protect PSI and PSII. Therefore, when photosynthesis was restricted in *V. planifolia*, modulation of photoprotection was mainly regulated by *g*_H_^+^ rather than CEF. This strategy is similar to C3 plants but is different from C4 plants.

## Data Availability

The data presented in this study are available on request from the corresponding author.
